# Application of Machine Learning Algorithms in Urinary Tract Infections Diagnosis Based on Non-Microbiological Parameters

**DOI:** 10.3390/pathogens14101034

**Published:** 2025-10-12

**Authors:** M. Mar Rodríguez del Águila, Antonio Sorlózano-Puerto, Cecilia Bernier-Rodríguez, José María Navarro-Marí, José Gutiérrez-Fernández

**Affiliations:** 1 Servicio de Medicina Preventiva y Salud Publica, Hospital Universitario Virgen de las Nieves, 18014 Granada, Spain; 2 PhD Program in Clinical Medicine and Public Health, University of Granada, 18016 Granada, Spain; asp@ugr.es; 3 Department of Microbiology, School of Medicine, University of Granada, 18016 Granada, Spain; 4 Instituto de Investigacion Biosanitaria ibs, 18012 Granada, Spain; 5 Faculty of Medicine, University of Almeria, 04120 Almeria, Spain; cbr968@inlumine.ual.es; 6 Laboratorio de Microbiologia, Hospital Universitario Virgen de las Nieves, 18014 Granada, Spain

**Keywords:** urinary tract infections, machine learning, diagnostic techniques and procedures, laboratory markers, algorithms

## Abstract

Urinary tract infections (UTIs) are among the most common pathologies, with a high incidence in women and hospitalized patients. Their diagnosis is based on the presence of clinical symptoms and signs in addition to the detection of microorganisms in urine trough urine cultures, a time-consuming and resource-intensive test. The goal was to optimize UTI detection through artificial intelligence (machine learning) using non-microbiological laboratory parameters, thereby reducing unnecessary cultures and expediting diagnosis. A total of 4283 urine cultures from patients with suspected UTIs were analyzed in the Microbiology Laboratory of the University Hospital Virgen de las Nieves (Granada, Spain) between 2016 and 2020. Various machine learning algorithms were applied to predict positive urine cultures and the type of isolated microorganism. Random Forest demonstrated the best performance, achieving an accuracy (percentage of correct positive and negative classifications) of 82.2% and an area under the ROC curve of 87.1%. Moreover, the Tree algorithm successfully predicted the presence of Gram-negative bacilli in urine cultures with an accuracy of 79.0%. Among the most relevant predictive variables were the presence of leukocytes and nitrites in the urine dipstick test, along with elevated white cells count, monocyte count, lymphocyte percentage in blood and creatinine levels. The integration of AI algorithms and non-microbiological parameters within the diagnostic and management pathways of UTI holds considerable promise. However, further validation with clinical data is required for integration into hospital practice.

## 1. Introduction

Urinary tract infections (UTIs), both complicated and uncomplicated, are highly prevalent in the general population, with a higher incidence in women, children under two years old, catheterized individuals, and patients with diabetes mellitus [1]. Globally, in 2019, the age-standardized incidence rate was 404.61 UTI cases per million inhabitants, with 30.12 cases per million in North America and 31.83 cases per million in Western Europe [2]. In Spain, the 2024 Nosocomial Infection Prevalence Study (EPINE) estimated the prevalence of UTIs at 20.13% among hospitalized patients, with *Escherichia coli* being the most frequently isolated microorganism [3]. In our local setting, prevalence rises to 23.53%, with *E. coli* responsible for 26.67% of UTIs [4].

The diagnosis of UTIs is based on the presence of clinical symptoms and signs in addition to the detection of microorganisms in urine trough urine cultures. In the era of Artificial Intelligence (AI), new opportunities arise to develop tools that optimize healthcare resource utilization, reduce diagnostic test turnaround times and associated costs, and enhance patient safety and satisfaction. AI not only improves outcome prediction by adapting existing resources to clinical demands but also enables the detection of potential anomalies in patient care processes. These techniques foster new approaches to information management in general and UTI diagnostics in particular, which would be unfeasible with conventional methods. AI facilitates the integration of multiple real-time data sources, aiding in both diagnosis and treatment decision-making [5].

The application of AI in disease diagnostics represents a significant breakthrough in modern medicine, enabling faster, more accurate, and more efficient diagnoses. Algorithms such as Random Forest, artificial neural networks, and gradient boosting have demonstrated their ability to identify complex patterns in clinical and laboratory data, surpassing traditional methods in sensitivity and specificity [6]. Additionally, AI-driven applications allow for the processing and analysis of structured information, such as anonymized clinical data, through statistical models or machine learning approaches that complement medical decision-making. These tools offer predictive insights, prioritize cases based on input data, and support evidence-based clinical decisions [7]. However, AI remains in an exploratory phase, requiring further studies analyzing large, heterogeneous, and prospective datasets to reach full-scale implementation [8]. While promising, these advancements necessitate additional validation before being fully integrated into routine clinical practice [9].

UTIs are a common pathology that demands substantial healthcare resources for diagnosis. AI models could not only optimize UTI detection but also reduce unnecessary or duplicated urine cultures and alleviate laboratory workloads. The diagnostic process could potentially be streamlined through automated algorithms and advanced machine learning techniques, which allow for UTI modeling while also identifying correlations with other analytical parameters. The objective of this study was to define, apply, and validate different classification algorithms to model UTI diagnosis and identify the most frequently implicated microbial groups using machine learning techniques based on non-microbiological laboratory parameters.

## 2. Materials and Methods

### 2.1. Population, Sample and Source of Information

A cross-sectional descriptive study was conducted at University Hospital Virgen de las Nieves (Granada, Spain), a reference healthcare center serving a population of approximately 334,000 inhabitants. The hospital houses several specialized laboratories, including Microbiology, Hematology, and Biochemistry. The study population consisted of patients with suspected UTIs referred from Specialized Care, including hospitalized patients as well as those from Outpatient Clinics and the Emergency Department.

A case was defined as any urine culture performed in the Microbiology Laboratory between January 2016 and December 2020, regardless of patient age or sex, provided that documented results from a urine dipstick test and basic hematological parameters (red blood cell, white blood cell, and platelet counts) were available (Laboratoire Sysmex, Sant Just Desvern, Barcelona, Spain). Urine cultures were excluded if the sample origin was unclear or if patient age and sex were not recorded.

Urine sample culturing followed the laboratory’s standardized protocol. A calibrated 1-μL inoculating loop was used, and samples were cultured on UriSelect4 chromogenic medium (Bio-Rad, Barcelona, Spain) and incubated at 36 ± 1 °C for 24 h. For patients attended in the Nephrology Department, an additional blood agar plate (BD, Madrid, Spain) was incubated in CO_2_. Colony counts were evaluated using the following cut-off values: in urine samples obtained by spontaneous miction or from a permanent catheter, UTI was defined by a count of ≥100,000 CFU (colony forming units)/mL or count of >10,000 CFU/mL for a single microorganism; in urine samples obtained from a temporary urethral catheter a count of ≥10,000 CFU/mL for one or two microorganisms was considered significant, and urine cultures with the growth of >2 microorganisms were excluded [10,11]. Microorganisms were subsequently identified using MALDI-TOF mass spectrometry (Biotyper, Bruker Daltonics, Billerica, MA, USA) and/or the MicroScan WalkAway system (Beckman-Coulter, Brea, CA, USA).

### 2.2. Variables and Statistical Analysis

The response variables were positive urine culture (yes/no) and the type of isolated microorganism (Gram-negative bacillus, Gram-positive coccus, yeast, others), later dichotomized as Gram-negative bacillus/others. The independent variables included were those indicated in Table 1. Only hematological and biochemical variables with less than 10% missing values were included in the analysis.

The statistical analysis followed several steps:

1. Descriptive Analysis: Qualitative variables were summarized using frequencies and percentages, while quantitative variables were described using means and standard deviations (for symmetric distributions) or medians and interquartile ranges (for asymmetric distributions).
2. Estimation of the Percentage of Positive Urine Cultures (positive urine cultures divided by the total number of urine cultures). This was accompanied by a 95% confidence interval.
3. Bivariate Analysis: Associations between quantitative variables and urine culture results were tested using Student’s *t*-test (for equal variances) or Welch’s *t*-test (for unequal variances). Associations with qualitative variables were assessed using Pearson’s chi-square test. Statistical significance was set at *p* < 0.05.
4. Machine Learning Algorithm Application:
  - Variable Recoding: All categorical variables were previously converted to dichotomous (positive-negative), except for months and sample type that were recoded as dummy variables.
  - Dataset Splitting: The dataset was divided into a training set (80%) for model development and a test set (20%) for validation, ensuring sufficient data for both stages to enhance model generalizability [12].
  - Missing Data Imputation: Missing values were imputed in the training set using the k-Nearest Neighbors (kNN) method, which identifies the k closest neighbors based on dataset variables and imputes missing values using the mean. Subsequently, imputation was performed on the test set using the imputation model learned on the training set.
  - Variable Normalization: To ensure all variables contributed equally to the model and to mitigate differences in measurement scales, quantitative values were rescaled between 0 and 1.
  - Pearson Correlation Analysis: Highly correlated variables (r > 0.7) were removed to reduce redundancy.
  - Variable Importance Assessment: The most relevant variables for the training set were selected using the Random Forest method with cross validation with 10 folds.
  - Machine Learning Algorithm Implementation: The following widely used algorithms were applied (the cross-validation method with 10 iterations of resampling was used for training). All the algorithms were initialized with the same seed to avoid reproducibility:
    ○ Logistic Regression—A classification model predicting binary outcomes using a logistic function.
    ○ k-Nearest Neighbors (kNN)—Classifies a data point based on the majority class of its k nearest neighbors using a distance metric.
    ○ Decision Trees—Splits data into successive decisions based on feature values, forming a tree structure.
    ○ Support Vector Machine (SVM)—Identifies the hyperplane that maximizes class separation.
    ○ Random Forest—An ensemble method averaging predictions from multiple decision trees.
    ○ Gradient Boosting Machine (GBM)—A sequential model where each iteration corrects the errors of the previous one.
    ○ Artificial Neural Network (NNet)—A multi-layered model that processes data hierarchically through interconnected nodes (neurons).
  - Model Prediction and Validation: Performance was evaluated using a confusion matrix (true positives, true negatives, false positives, and false negatives) in the test set.
  - Accuracy Calculation: Defined as the proportion of correct predictions over the total number of predictions.
  - Sensitivity, Specificity, and ROC Curve Analysis: Performance comparisons among models were conducted using receiver operating characteristic (ROC) curves, with sensitivity and specificity reported alongside 95% confidence intervals.

The statistical analysis was conducted using RStudio (version 2023.06.1+524), and the machine learning algorithms were implemented via the “caret”package (version 7.0.1) in R. The caret package (classification and regression training) includes functions to perform complex methods for classification and regression of variables (https://topepo.github.io/caret/index.html) (accessed on 3 February 2025).

### 2.3. Ethical Considerations

Urine cultures and analytical parameters were obtained as part of standardized hospital procedures and were not linked to additional research interventions. Consequently, as this was a retrospective study, informed patient consent was not required for data analysis. Patient records were anonymized using an identification number, and data were processed in aggregate to prevent patient identification. The study received approval from the provincial ethics committee on 21 December 2020 (reference code 1671-N-20). A data management plan was developed to define the life cycle of data, its processing and management in accordance with EU Regulation 2016/679 on the protection of individuals with regard to the processing of personal data (GDPR).

## 3. Results

Between January 2016 and December 2020, a total of 6747 urine samples for urine culture were received in the Microbiology laboratory, of which 66 were discarded due to coding issues. After verifying the availability of documented results from a urine dipstick test, as well as hematological and biochemical results, and excluding cases with more than 10% missing values, the final sample consisted of 4283 urine cultures. Of the 42 variables included in the study, 36 had a range of missing values between 0.3% and 7.5%.

Table 2 presents the values obtained for the study variables, expressed as mean ± standard deviation for quantitative variables and as percentages for qualitative variables. The mean age of the patients was 56.4 ± 23.1 years. Of the total urine cultures, 4108 (95.9%) were from adults (aged 18 years or older), and 51.5% were from female patients. The majority of samples were collected as spontaneous miction (83.8%), during January (9.6%), and originated from community settings (emergency services and outpatient clinics) in 91.9% of cases. The proportion of urine cultures with a glucose level of 0 mg/dL was 90.4%, while ketone bodies, proteins, and nitrites had values of 0 mg/dL in 80.0%, 44.2%, and 83.1% of cases, respectively. Additionally, the absence of red and white blood cells was observed in 29.8% and 36.4% of the samples, respectively.

A total of 38.6% of urine cultures were positive (95% CI: 37.2–40.1%), with a single microorganism isolated in 86.8% of these cases and two microorganisms in the remaining cases. Gram-negative bacilli were identified in 68.8% of positive cultures, with *E. coli* being the most frequently isolated bacterium, while Gram-positive cocci were found in 20%, with *Enterococcus faecalis* being the most prevalent species. In the bivariate analysis (Table 2), significant differences were observed between positive and negative urine cultures for all study variables (*p* < 0.05), except for the month of sample collection, glucose, bilirubin, and ketone bodies in the urine dipstick test, as well as lymphocyte count, mean corpuscular and platelet volume in the red and platelet series, and the hemolytic, icteric, and lipemic indices in the hematological analysis.

Among positive urine cultures, higher values were observed for patient age, female sex, samples collected during the summer months and December, those obtained from permanent and temporary urethral catheterization, and those of community origin. Significantly higher values were also found for pH, hematuria (≥2+), protein levels (≥1 mg/dL), the presence of nitrites, leukocytes (≥3+), creatinine, basophil count, immature granulocyte count and percentage, leukocyte count, lymphocyte percentage, monocyte count, neutrophil count and percentage, platelet count, and red cell distribution width. The remaining variables were significantly higher in negative urine cultures.

The dataset was split into 80% training data (3427 records) and 20% test data (856 records), with both subsets maintaining a positive urine culture rate of 38.6%, identical to the full dataset.

**Figure 1:**
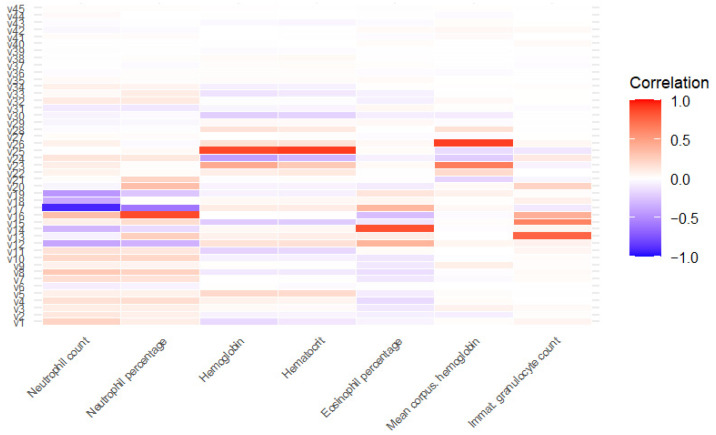
Correlation between highly correlated variables and the rest of variables. v1: age; v2: glucose; v3: bilirrubin; v4: ketone bodies; v5: density; v6: pH; v7: hematuria; v8: proteins; v9: urobilinogen; v10: leukocytes; v11: creatinine; v12: basophil percentage; v13: basophil count; v14: eosinophil count; v15: immature granulocyte percentage; v16: white blood cell count; v17: lymphocyte percentage; v18: lymphocyte count; v19: monocyte percentage; v20: monocyte count; v21: platelet count; v22: mean platelet volume; v23: mean corpuscular hemoglobin concentration; v24: red cell distribution with index; v25: red blood cell count; v26: mean corpuscular volume; v27: hemolytic index; v28: icteric index; v29: lipemic index; v30: sex; v31: origin; v32: nitrites; v33: temporary urethral catheter v34: permanent catheter; from v35 to v45: January to November.

A correlation analysis (Figure 1) identified seven variables (neutrophil count, neutrophil percentage, hemoglobin, hematocrit, eosinophil percentage, mean corpuscular hemoglobin and immature granulocyte count) that were highly correlated with others (significant correlation >0.7) and were excluded from subsequent analyses. After applying the variable importance algorithm using “rfe” with Random Forest, all variables were selected as important, indicating that none were discarded due to lack of relevance in the model.

The seven selected machine learning algorithms were trained on the training set with the following parameters: k = 9 in kNN, mtry = 2 in RF, sigma = 0.01530869 and C = 0.5 in SVM, n.trees = 150, interaction.depth = 3, shrinkage = 0.1 and n.minobsinnode = 10 in GBM. The percentage of minority group values was almost 39%, therefore it was not considered an unbalanced study. After applying them to the test set for validation, the Random Forest and Gradient Boosting Machine algorithms demonstrated the best performance, with accuracies of 82.2% and 78.2%, respectively. Meanwhile, the Support Vector Machine algorithm, neural network and logistic regression yielded more modest results, with accuracies around 77%. The decision tree models and kNN also performed well overall, although their accuracy was slightly lower (Table 3).

**Figure 2:**
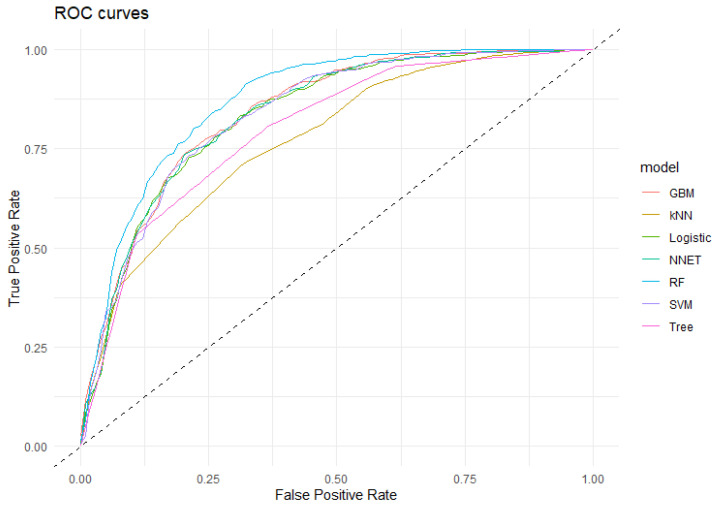
ROC curve of the algorithms applied in the detection of positive urine cultures. GBM: Gradient Boost Machine; kNN: k Nearest Neighbourg; Logistic: Logistic Regresion; NNet: Neural Network; RF: Random Forest; SVM: Support Vector Machine; Tree: Decision Tree. Dashed line: baseline.

The ROC curves (Figure 2) generated for each model generally indicated high discriminative power, with an area under the curve (AUC) exceeding 87% for Random Forest and over 83% for Gradient Boost Machine, Support Vector Machine, Neural Network and Logistic Regression (Table 3). In comparison, the kNN and Decision Tree models had lower AUC values, suggesting a reduced ability to distinguish between positive and negative urine cultures.

The analysis of variable importance in the Random Forest model, which had the highest discriminative power, identified the most influential predictors of positive urine cultures as the presence of leukocytes and nitrites and density in the urine dipstick test, along with elevated white cells count, monocyte count, lymphocyte percentage in blood and creatinine levels. Conversely, the least important variables were sample origin, glucose and month of year.

Among the positive urine culture samples (n = 1655), the isolated microorganism variable was dichotomized into two groups: Gram-negative bacilli and others. The percentage of isolates in the Gram-negative bacilli group was 66.8% (CI95%: 64.6–69.1%), mostly located in the first isolate. A bivariate analysis was performed to compare predictive variables, identifying 18 significant variables. Lower mean values were observed in the Gram-negative bacilli group for age, number of isolation, basophil percentage, lymphocyte percentage, monocyte percentage, eosinophil count and percentage, red cell distribution width, hospital origin, male sex, and urine samples obtained via permanent bladder catheterization (*p* < 0.05).

After imputing missing values to the training sample and subsequently eliminating the most correlated variables, the variable importance procedure was applied to determine the most significant variables for the application of the algorithms. The resulting variables are the 15 indicated in Table 4.

The seven machine learning algorithms were trained in the train set and applied to the test set to predict whether the isolated microorganism was a Gram-negative bacillus. The estimated parameters were as follows: k = 5 in kNN, mtry = 8 in RF, sigma = 0.06761723 and C = 0.25 in SVM, n.trees = 50, interaction.depth = 2, shrinkage = 0.1 and n.minobsinnode = 10 in GBM. The best predictive performance was achieved by the Tree algorithm (77.3% accuracy), with a sensitivity of 90.5% (Table 4). Neural Network algorithm, GBM algorithm, Logistic Regression and Support Vector Machine provided the same accuracy (76.4%) and similar sensitivities (86–92%). The most important predictive variables in this model were number of isolation, red cell distribution width index, eosinophil count and monocyte count, while origin, proteins (dipstick test) and mean platelet volume were the least important.

## 4. Discussion

The present study highlights the advantage of applying machine learning algorithms to a set of urine samples to automate the detection of positive urine cultures solely based on laboratory parameters. The study demonstrated that the Random Forest algorithm yielded the most satisfactory results, with an area under the ROC curve (AUC) of 87.10%, showing good specificity in detecting negative urine cultures (92.76%) and moderate sensitivity for detecting positive cases (65.56%). The novelty of the study is that only laboratory parameters were used without including other types of clinical variables, risk factors, patient history or treatments, a fact that has been extensively studied by most of the existing publications on the subject that were found, which are cited in this article. Biochemical and hematological variables scarcely studied in the literature have been included, none of them in our geographical area, with a high prevalence of UTI, which adds relevance and interest to the findings, since with a more limited set of data, results similar to some of the published studies that do consider them are obtained.

Various studies have applied machine learning techniques for UTI detection based on different variables. A study conducted in the United Kingdom, where UTIs were one of the five leading causes of hospital admissions (accounting for approximately 9%), reported an accuracy of 85% in correctly classifying UTIs using variables related to sleep patterns [13]. Ozkan et al. developed an algorithm with 98% accuracy for diagnosing UTIs based on medical history, clinical examination, and ultrasound techniques [14]. Aydin et al. found an accuracy of 91% in a pediatric study, linking UTIs with the presence of nitrites, leukocytes, catheterization, blood samples, and gender [15]. The work by Gadalla et al. identified a model in women using clinical variables, biomarkers, and urine turbidity, achieving 70% accuracy [16]. In an emergency department setting, a maximum accuracy of 87% was obtained [17]. Finally, a study with a larger sample size than the previous ones predicted UTIs with 98% accuracy using hemogram variables, along with patient age and gender [18].

In hospitalized geriatric patients, a relationship has been found between UTIs and bloodstream bacteremia, after evaluating the utility of parameters such as white blood cell count, creatinine concentration, and erythrocyte sedimentation rate (ESR), which significantly increase infection severity. Bacteremia can lead to severe complications requiring more intensive care. This underscores the importance of accurately assessing laboratory parameters in the management of UTIs in older adults [19]. A correlation has also been observed between hematological parameters and urine culture results in pregnant women, with higher white blood cell levels in cases of asymptomatic bacteriuria [20]. In patients with permanent urinary catheters, the presence of leukocytes and red blood cells in urine may reflect colonization rather than true infection. Differentiating between these conditions requires a thorough clinical assessment, including evaluation of symptoms, inflammatory markers, and response to therapy, variables not addressed in the present study. Therefore, our findings, based solely on laboratory parameters, should be interpreted with caution in this subgroup, and always in conjunction with clinical judgment to avoid potential misclassification.

Although urine density showed statistically significant differences between groups, the absolute difference was minimal (1.017 vs. 1.018) and likely lacks clinical relevance. This finding, while statistically robust due to the large sample size, should be interpreted with caution, as it does not reflect a physiologically meaningful difference in the context of urinary tract infection diagnosis. On the other hand, the non-linear distribution of hematuria, proteinuria, and leukocyturia observed in this study may reflect the influence of multiple coexisting conditions and the limitations of single-marker interpretation. Mild alterations may be related to non-infectious etiologies, while higher grades are more often associated with true urinary tract infections. These findings highlight the importance of interpreting urinalysis results in the broader clinical context and avoiding over-reliance on isolated parameters.

The present study shows similar results to those reported by Taylor [17] who, using 37 variables, demonstrated that the Random Forest algorithm was the most accurate, with an AUC of 87.4%. The main difference between both studies is that Taylor’s included variables from medical history, as well as patient signs and symptoms. Similarly, in the study by Nyman [21], the Random Forest algorithm stood out among the applied methods, reducing the workload of cultures by 46% by identifying negative samples, while maintaining a sensitivity of 95% and a specificity of 72%, based on 33 predictive variables derived from flow cytometry. Likewise, Dedeené [22] identified a logistic regression model using flow cytometry parameters, achieving an AUC of 85.8%, whereas applying a neural network resulted in an AUC of 88.4%. Del Ben [23] developed a machine learning model based on decision trees to improve urine sample screening and reduce microbiology laboratory workload. The model achieved a sensitivity of 94.5% and allowed for the identification of negative samples, further reducing unnecessary cultures by 16%, with an estimated financial impact of EUR 40,000 annually. Although our study obtained more moderate sensitivity values, in terms of accuracy they are comparable to those cited, with the advantage of having analyzed only laboratory variables, which are easier to obtain in our environment than clinical variables. These findings suggest that applied methodologies have the potential to optimize clinical diagnostic processes, saving both time and significant costs.

Regarding the prediction of the microorganism responsible for the UTI, our results suggest that five of the seven algorithms used (Tree, Neural Network, GBM and SVM) can be applied to predict that the causative agent belongs to the Gram-negative bacilli group (including *E. coli*). Using these algorithms, an AUC of 78.5–82.1% was obtained, with a sensitivity around 90% in all cases, although with moderate specificity. Our figures are slightly higher than those reported by Choi [24,25], who obtained an AUC of approximately 74% using automated urine tests, while AI models, particularly XGBoost, achieved a high ROC AUC (>90%). It should be noted that in Choi’s study, samples from more than 50,000 patients were used, about ten times more than those included in this study, providing much greater statistical power and consistency to their results. Machine learning algorithms are specifically designed to work with large datasets; although they can function with smaller ones, they may overfit the training data, reducing their ability to generalize to new cases. Additionally, the variability in smaller datasets may be insufficient to capture complex patterns [26]. Despite including a small sample in our study, the high sensitivity values obtained show that the algorithms could be used as a preliminary screening tool in the detection of Gram-negative bacilli, including *E. coli*.

With regard to the number of isolates in positive ITUs, some studies show that the isolation of two microorganisms is often labeled as contamination and therefore many models exclude them. At the methodological level, there are studies that specifically predict mixed growth as a sign of contamination, showing that it is an important source of false positives if not modeled separately. At the same time, recent reviews highlight that some polymicrobial cultures may represent real infections and that systematically excluding them can introduce bias and lead to the loss of clinically relevant cases, so they propose reconsidering their inclusion in the diagnosis [27]. In our study, most monomicrobial cultures were caused by Gram-negative bacilli. Likewise, in polymicrobial cultures, at least one of the isolates was a Gram-negative bacillus, supporting their predominant role in urinary tract infections and highlighting their relevance in the prediction of these pathogens. Microbiological confirmation of the causative microorganism and its antimicrobial susceptibility profile is essential for the rational selection of antibiotic therapy, prevention of resistance, and individualized management of urinary tract infections. The use of the proposed algorithms should always be complemented by microbiological and clinical interpretation, ensuring maximum efficacy and safety for the patient.

Beyond this limitation, the main drawback of the present study is the exclusive consideration of laboratory variables for developing the algorithms. The hospitalization and emergency information systems at our hospital are separate from the laboratory systems, making it highly complex to cross-reference databases for obtaining other types of clinical or hospital admission variables. Nevertheless, very satisfactory results have been obtained, which would likely have been even better had we been able to complement the study with these additional variables. According to clinical guidelines, the first urine sample obtained in suspected urinary tract infection should be used for both biochemical analysis and urine culture, ensuring proper collection technique to minimize contamination. Burton demonstrated that by applying machine learning algorithms, laboratory workload can be reduced by up to 41% [17].

The algorithms found have shown satisfactory results for the overall sample, but when particularized to positive urine cultures, they have turned out to be very modest. Therefore, it is necessary to expand the sample and/or set of predictor variables in order to achieve more satisfactory predictions in this group.

The main strength, on the other hand, is that by predicting the positivity or negativity of urine cultures using only laboratory parameters obtained from routine work, the cost of the determinations is affordable, and the necessary information for making predictions is much simpler and faster to collect.

Exploratory models based on machine learning have proven to be highly valuable tools in the field of medical diagnostics in general and in the detection of positive urine cultures in particular. These models allow for the analysis of large volumes of clinical and/or laboratory data, identifying patterns that might go unnoticed with traditional approaches. By learning from historical data, algorithms can improve the accuracy of UTI detection by evaluating multiple variables simultaneously, optimizing the diagnostic process, reducing false negatives, and facilitating earlier intervention. The ability to adjust models to local datasets or specific populations further enhances their utility, as they can be tailored to particular patient characteristics, improving personalized treatment.

Finally, the integration of AI algorithms and non-microbiological parameters within the diagnostic and management pathways of UTI holds considerable promise. Nevertheless, it is imperative to acknowledge that such tools must be regarded as complementary rather than substitutive, as microbiological confirmation of the causative pathogen and its antimicrobial susceptibility remains the cornerstone of safe and rational management. The advancement of this field is contingent upon the integration of innovative computational models with conventional microbiological methodologies. This integration will promote the development of more personalized, efficient, and evidence-based clinical strategies.

## 5. Conclusions

The use of machine learning models has proven effective in predicting UTIs with high-moderate sensitivity and specificity, based on laboratory parameters. The presence of leukocytes and nitrites in the urine dipstick test, along with elevated white cells count, monocyte count, lymphocyte percentage in blood and creatinine levels, were identified as key markers for diagnosing these infections. These models are easy to implement in healthcare settings, as they can be integrated into routine clinical software applications, providing clinicians with a valuable tool to diagnose UTIs and determine their most likely etiology.

This study is exploratory, as a first step towards the detection of UTIs using machine learning models, so the conclusions are an advance in the application of these models. The interpretations of these results should be made with caution, in the sense that confirmatory studies with more samples and more variables are needed to validate the results obtained.

## Figures and Tables

**Table 1:** List of the 42 variables included in the study.

Independent Variables	White Blood Cell Series (WBC)
Age (years) *†	White blood cell count *†
Sex (male, female) *	Neutrophil count
Sample type (spontaneous miction, permanent catheter, temporary urethral catheter) *	Neutrophil percentage
Origin (hospital, community) *†	Lymphocyte count *†
Number of isolations (only in positive urine cultures) *†	Lymphocyte percentage *†
Month of urine culture collection (January to December) *	Monocyte count *†
**Variables from urine dipstick test**	Monocyte percentage *
Glucose (mg/dL) *	Basophil count *
Bilirubin *	Basophil percentage *†
Ketone bodies (mg/dL) *	Eosinophil count *†
Density *	Eosinophil percentage
Ph *	Immature granulocyte count
Hematuria (negative, +, ++, +++, 4+ and 5+) *†	Immature granulocyte percentage *
Proteins (mg/dL) *†	**Platelet Series (PS)**
Urobilinogen (mg/dL) *	Platelet count *
Nitrites (negative, positive) *†	Mean platelet volume *†
Leukocytes (negative, +, ++, +++, 4+ and 5+) *†	**Other Blood Parameters**
**Red Blood Cell Series (RBC)**	Creatinine *
Red blood cell count *	Hemolytic index *
Mean corpuscular volume *	Icteric index *
Red cell distribution width index *†	Lipemic index *
Hematocrit	
Hemoglobin	
Mean corpuscular hemoglobin	
Mean corpuscular hemoglobin concentration *	

* Variables included in the application of machine learning algorithms for the determination of positive urine cultures. † Variables included in the prediction of Gram-negative bacilli. + Positive +. ++ Positive 2+. +++ Positive 3+

**Table 2:** Values obtained for all study variables, bivariate analysis according to positive or negative urine culture, and results of the comparison tests for two samples (Student’s *t*-test, Welch’s test, and Chi-square test).

Variable	n	Total Sample	Positive Urine Culture (38.6%)	Negative Urine Culture (61.4%)	*p*-Value
**Independent variables**					
Age	4283	56.4 ± 23.1	59.0 ± 24.0	54.7 ± 22.4	<0.001
Sex (woman)	4283	2204 (51.5)	951 (57.5)	1253 (47.7)	<0.001
Spontaneous miction	4283	3589 (83.8)	1271 (76.8)	2318 (88.2)	<0.001
Temporary urethral catheter		484 (11.3)	246 (14.9)	238 (9.1)	
Permanent catheter		210 (4.9)	138 (8.3)	72 (2.7)	
Origin (hospital)	4283	347 (8.1)	112 (6.8)	235 (8.9)	<0.05
January	4283	412 (9.6)	138 (8.3)	274 (10.4)	0.068
February		408 (9.5)	162 (9.8)	246 (9.4)	
March		370 (8.6)	140 (8.5)	230 (8.8)	
April		379 (8.8)	139 (8.4)	240 (9.1)	
May		390 (9.1)	145 (8.8)	245 (9.3)	
June		388 (9.1)	143 (8.6)	245 (9.3)	
July		319 (7.4)	130 (7.9)	189 (7.2)	
August		293 (6.8)	126 (7.6)	167 (6.4)	
September		321 (7.5)	149 (9.0)	172 (6.5)	
October		359 (8.4)	134 (8.1)	225 (8.6)	
November		361 (8.4)	134 (8.1)	227 (8.6)	
December		283 (6.4)	115 (6.9)	168 (6.4)	
**Urine dipstick test**					
Glucose	4272				0.275
0 mg/dL		3863 (90.4)	1474 (89.3)	2389 (91.1)	
1–50 mg/dL		139 (3.3)	61 (3.7)	78 (3.0)	
51–100 mg/dL		85 (2.0)	35 (2.1)	50 (1.9)	
>100 mg/dL		185 (4.3)	80 (4.9)	105 (4.0)	
Bilirrubin	4270	0.2 ± 0.6	0.24 ± 0.56	0.23 ± 0.58	0.643
Ketone bodies	4269				0.267
0 mg/dL		3415 (80.0)	1297 (78.7)	2118 (80.8)	
1–20 mg/dL		643 (15.1)	274 (16.6)	369 (14.1)	
21–40 mg/dL		19 (0.4)	7 (0.4)	12 (0.5)	
41–60 mg/dL		109 (2.6)	41 (2.5)	68 (2.6)	
>60 mg/dL		83 (1.9)	30 (1.8)	53 (2.0)	
Density	3962	1.018 ± 0.007	1.017 ± 0.007	1.018 ± 0.008	<0.001
pH	4270	6.01 ± 0.95	6.06 ± 0.97	5.98 ± 0.94	<0.05
Hematuria *	4249				<0.001
Negative		1267 (29.8)	268 (16.3)	999 (38.3)	
Positive +		1111 (26.1)	412 (25.1)	699 (26.8)	
Positive 2+		385 (9.1)	164 (10.0)	221 (8.5)	
Positive 3+		469 (11.0)	260 (15.8)	209 (8.0)	
Positive 4+ and 5+		1017 (23.9)	540 (32.8)	477 (18.3)	
Proteins	4270				<0.001
0 mg/dL		1887 (44.2)	498 (30.2)	1389 (53.0)	
1–50 mg/dL		1254 (29.4)	514 (31.2)	740 (28.2)	
51–100 mg/dL		617 (14.4)	348 (21.1)	269 (10.3)	
>100 mg/dL		512 (12.0)	289 (17.5)	223 (8.5)	
Urobilinogen	4270	0.42 ± 1.34	0.36 ± 1.20	0.46 ± 1.42	<0.05
Nitrites (positive)	4270	722 (16.9)	576 (34.9)	146 (5.6)	<0.001
Leukocytes *	4270				<0.001
Negative		1555 (36.4)	202 (12.2)	1353 (51.6)	
Positive +		64 (1.5)	22 (1.3)	42 (1.6)	
Positive 2+		681 (15.9)	168 (10.2)	513 (19.6)	
Positive 3+		595 (13.9)	266 (16.1)	329 (12.6)	
Positive 4+ and 5+		1375 (32.2)	991 (60.1)	384 (14.7)	
**Red blood Cell Series (RBC)**					
Red blood cell count		4.44 ± 0.75	4.35 ± 0.73	4.49 ± 0.74	<0.001
Mean corpuscular volumen	4218	88.43 ± 7.24	88.36 ± 7.69	88.47 ± 6.94	0.636
Red cell distribution width index	4215	14.19 ± 2.24	14.37 ± 2.31	14.07 ± 2.18	<0.001
Hematocrit	4218	39.08 ± 6.19	38.31 ± 6.13	39.57 ± 6.19	<0.001
Hemoglobin	4218	13.00 ± 2.20	12.69 ± 2.18	13.19 ± 2.20	<0.001
Mean corpuscular hemoglobin	4218	29.39 ± 2.77	29.26 ± 2.89	29.48 ± 2.69	<0.05
Mean corpuscular hemoglobin concentration	4218	33.20 ± 1.83	33.04 ± 2.02	33.30 ± 1.70	<0.001
**White Blood Cell Series (WBC)**					
White blood cell count	4218	10.30 ± 6.18	11.6 ± 6.96	9.47 ± 5.48	<0.001
Neutrophil count	4218	7.55 ± 5.01	8.73 ± 5.29	6.81 ± 4.68	<0.001
Neutrophil percentage	4218	70.23 ± 15.14	73.04 ± 14.70	68.45 ± 15.14	<0.001
Lymphocyte count	4218	1.77 ± 2.29	1.81 ± 3.25	1.75 ± 1.38	0.465
Lymphocyte percentage	4218	19.72 ± 12.59	17.28 ± 12.11	21.26 ± 12.65	<0.001
Monocyte count	4218	0.80 ± 1.05	0.89 ± 0.79	0.75 ± 1.18	<0.001
Monocyte percentage	4218	8.06 ± 4.23	7.85 ± 4.08	8.20 ± 4.32	<0.05
Basophil count	4218	0.04 ± 0.04	0.04 ± 0.06	0.04 ± 0.02	<0.01
Basophil percentage	4218	0.42 ± 0.30	0.40 ± 0.31	0.44 ± 0.29	<0.001
Eosinophil count	4218	0.11 ± 0.15	0.10 ± 0.13	0.12 ± 0.16	<0.001
Eosinophil percentage	4218	1.36 ± 1.70	1.14 ± 1.48	1.51 ± 1.81	<0.001
Immature granulocyte count	4050	0.11 ± 0.66	0.15 ± 0.99	0.08 ± 0.30	<0.01
Immature granulocyte percentage	4058	0.80 ± 1.78	0.91 ± 2.17	0.74 ± 1.47	<0.01
**Platalet Series (PS)**					
Platelet count	4218	241.37 ± 102.50	250.45 ± 109.90	235.63 ± 97.12	<0.001
Mean platelet volumen	4139	10.69 ± 1.21	10.67 ± 1.21	10.69 ± 1.22	0.620
**Other blood parameters**					
Creatinine	4148	1.18 ± 1.05	1.24 ± 1.03	1.15 ± 1.07	<0.05
Hemolytic index	4209	5.0 [2.0–13.0]	5.0 [2.0–15.0]	5.0 [2.0–13.0]	0.125
Icteric index	4209	0.8 [0.6–1.1]	0.8 [0.5–1.1]	0.8 [0.6–1.2]	0.219
Lipemic index	4209	2.0 [0.0–6.0]	2.0 [0.0–6.0]	2.0 [0.0–6.0]	0.316

Results expressed as mean ± standard deviation, frequency (%) and median [interquartile range]. * Hematuria and Leukocytes are expressed in gradients of positivity.

**Table 3:** Application of the algorithms on the test set in the determination of positive urine cultures, according to decreasing order of accuracy.

Algorithm	Sensitivity	Specificity	Accuracy (CI95%)	Kappa	AUC (CI95%)
RF	65.56	92.76	82.24 (79.51–84.75)	60.90	87.10 (84.55–89.66)
GBM	62.24	88.19	78.15 (75.23–80.88)	52.26	84.08 (81.31–86.85)
SVM	59.21	89.52	77.80 (74.87–80.55)	51.02	83.45 (80.60–86.31)
NNet	60.12	88.76	77.69 (74.75–80.44)	50.96	83.60 (80.76–86.44)
LR	62.24	87.43	77.69 (74.75–80.44)	51.36	83.24 (80.36–86.11)
Tree	63.44	80.57	73.95 (70.87–76.86)	44.49	79.74 (76.79–82.69)
kNN	53.47	80.76	70.21 (67.02–73.26)	35.28	77.19 (74.04–80.35)

RF: Random Forest; GBM: Gradient Boost Machine; SVM: Support Vector Machine; NNet: Neural Network; LR: Logistic Regression; Tree: Decision Tree; kNN: k Nearest-Neighbourg; CI95%: 95% Confidence Interval; AUC: Area Under ROC Curve.

**Table 4:** Results of the application of the algorithms on the test set in the determination of Gram-negative bacilli according to decreasing order of accuracy (n = 1655).

Algorithm	Sensitivity	Specificity	Accuracy (CI95%)	Kappa	AUC (CI95%)
Tree	90.50	50.46	77.27 (72.37–81.68)	44.36	79.01 (74.23–83.79)
NNet	86.43	55.96	76.36 (71.40–80.84)	44.24	82.10 (77.42–86.78)
GBM	90.95	46.79	76.36 (71.40–80.84)	41.40	81.44 (76.62–86.27)
SVM	92.31	44.04	76.36 (71.40–80.84)	40.49	78.45 (73.23–83.67)
kNN	86.43	55.05	76.06 (71.08–80.56)	43.39	76.77 (71.50–82.05)
LR	91.86	42.20	75.45 (70.44–80.00)	38.04	81.38 (76.61–86.15)
RF	87.33	49.54	74.85 (69.81–79.44)	39.34	78.89 (73.79–84.00)

Tree: Decision Tree; NNet: Neural Network; GBM: Gradient Boost Machine; SVM: Support Vector Machine; kNN: k Nearest-Neighbourg; LR: Logistic Regression; RF: Random Forest; CI95%: 95% Confidence Interval; AUC: Area Under ROC Curve.

## Data Availability

The original contributions presented in this study are included in the article. Further inquiries can be directed to the corresponding author.

## Abbreviations

The following abbreviations are used in this manuscript:

UTI	Urinary Tract Infection
AI	Artificial Intelligent
EPINE	Nosocomial Infection Prevalence Study (Spain)
CFU	Colony Forming Units
RBC	Red Blood Cell Series
WBC	White Blood Cell Series
PS	Platelet Series
RF	Random Forest
SVM	Support Vector Machine
GBM	Gradient Boost Machine
NNet	Neural Network
LR	Logistic Regression
Tree	Decision Tree
kNN	k Nearest-Neighbourg
CI95%	95% Confidence Interval
ROC	Receiver Operating Characteristic curve
AUC	Area Under ROC curve
